# The association between perceived organizational support and academic job burnout: a comparative study of career stages and professional sectors

**DOI:** 10.3389/fpsyg.2026.1782137

**Published:** 2026-09-17

**Authors:** Juan Zhang, Xianghui Zhao

**Affiliations:** 1 The School of Public Administration, Southwestern University of Finance and Economics, Chengdu, China; 2 Institute of Educational Sciences, Hunan University, Changsha, Hunan, China

**Keywords:** academic profession, career stage, job burnout, perceived organizational support, professional sector

## Abstract

**Objective:**

Job burnout among academic professionals is a growing concern in organizational psychology, with important implications for employee wellbeing and the sustainability of knowledge-intensive work environments. This study examines the relationship between perceived organizational support and job burnout, and whether this relationship varies across career stages and professional sectors.

**Methods:**

Data were drawn from the Nature Career Job and Salary Satisfaction Survey 2021, a large-scale international survey of academic working conditions. The survey was administered in five languages and yielded 3,210 valid responses from academic professionals across 95 countries.

**Results:**

Three key findings emerge. First, burnout is widespread, with women, early-career researchers, and those in academia facing higher risks. Second, cross-national comparisons suggest heterogeneous patterns in which organizational support and job burnout do not always align. Third, organizational support is significantly associated with lower levels of burnout, though the strength and direction of this association vary across career stages and sectors.

**Implications:**

These findings suggest the need to view burnout as an early warning signal, establish structured support systems, and deliver targeted interventions to promote a more sustainable academic environment.

## 1 Introduction

The academic profession has traditionally been regarded as a vocation grounded in intellectual autonomy, intrinsic motivation, and the pursuit of knowledge. Yet in recent decades, this idealized image has been increasingly challenged. Under the expansion of new managerialism, marked by intensified performance measurement, accountability, and competition, academic work has become progressively characterized by heavy workloads, time scarcity, and growing psychological strain (Weinstein et al., 2023). As a result, job burnout has emerged as a widespread and persistent condition among academic professionals across national contexts (Asfahani, 2024; Xu, 2019). Large-scale surveys consistently show that academics report higher levels of burnout than many other occupational groups, with profound consequences for physical health, psychological well-being, and career sustainability (e.g., Jerrim et al., 2021; Edú-Valsania et al., 2022). Job burnout in academia has thus increasingly been recognized as a global and persistent concern. Burnout not only leads to physical health problems such as chronic fatigue, headaches, and hypertension (Edú-Valsania et al., 2022), but also undermines key psychological indicators, including job satisfaction, emotional well-being, and life happiness (Maddock, 2024).

In practice, efforts to alleviate academic burnout are realized primarily at the organizational level, where academics experience support or its absence in their everyday work (Menges, 1985). Despite growing recognition of academic burnout as a global problem, existing research has largely focused on its prevalence, outcomes, and individual-level determinants (e.g., Zhang et al., 2023; Miao et al., 2024). As a result, three issues remain insufficiently addressed. First, it is still unclear whether academic burnout follows common global patterns or varies systematically across national contexts. Second, although organizational support is frequently invoked as a relevant contextual factor, its association with academic burnout has rarely been examined in a systematic and cross-national manner. Third, limited attention has been paid to whether this association differs across career stages and professional sectors, despite substantial variation in academics’ work conditions and career structures.

Against these backdrops, the present study seeks to address the following research questions: What is the overall state and global pattern of job burnout in the academic profession? To what extent is perceived organizational support associated with academic job burnout? And does this association vary across career stages and professional sectors? Addressing these questions will yield empirical insights into how organizational support can be better structured to support academics’ well-being and professional development.

## 2 Literature review

### 2.1 The academic profession in a high-pressure environment

The academic profession is an autonomous occupation centered on scholarly work, characterized by specialized knowledge, professional responsibility, and adherence to academic norms and ethics (Carvalho and Diogo, 2018). Because academic work has historically been concentrated within universities, many studies have narrowly defined the academic profession as “university teachers” (De Frutos Belizon et al., 2025). However, with the transformation of knowledge production modes, the scope of the academic profession in the post-academic science era has expanded beyond universities to include all scholars engaged in academic activities across higher education institutions, research institutes, government agencies, and private enterprises (Ziman, 2000). This broader definition is adopted in the present study.

According to Weber (2012), the academic profession embodies both spiritual and material dimensions. Its spiritual dimension reflects a “calling,” viewing scholarship as a vocation devoted to the pursuit of knowledge and truth, whereas its “material” dimension emphasizes a career, treating scholarly work as a means of livelihood. For much of the twentieth century, the spiritual aspect predominated—scholars willingly dedicated themselves to established intellectual ideals and often imposed exacting standards on themselves. Baldwin (1990) observed that many accomplished academics voluntarily engaged in research and writing during their leisure time, thereby contributing to a high-workload culture that disdains idleness. Within this culture, academics seldom experienced burnout despite the heavy demands; rather, they often drew from their pursuits what Weber described as a “sacred spark” or what later sociologists called an “inner bliss” derived from the intrinsic rewards of scholarship (Rodgers and Rodgers, 1999).

However, with the growing influence of performance-based management and efficiency-driven evaluation systems, the academic profession has undergone profound changes—deteriorating work environments, declining professional status, intensified assessment requirements, heavier workloads, and insufficient organizational support (Siekkinen et al., 2020; Evans, 2022). Academics are increasingly compelled to devote excessive time and effort to meeting institutional expectations and ensuring career survival, facing the risk of marginalization if they fail to do so. A global survey conducted by *Nature* revealed that 12% of academic professionals lost their jobs amid funding-related layoffs, while 43% reported negative emotions toward their career prospects due to financial constraints, communication barriers, and disrupted research activities (Woolston, 2021a).

These findings indicate that academics today face dual pressures arising from a deeply ingrained high-workload culture and externally imposed performance management systems. This strain is reflected directly in their working hours: empirical evidence shows that academics routinely work over 50 h per week, and in many universities, workloads exceed 70 h (Bentley and Kyvik, 2012). Yet under the encroachment of external performance regimes, the intrinsic meaning of academic work has been eroded. The traditional high-workload culture no longer generates a sense of internal fulfillment; instead, it exposes academics to psychological exhaustion and distress. Berg and Seeber (2016) found that 78% of academics agreed that their work is long but ineffective, and 62% reported feeling physically and mentally drained every day—figures far higher than those of corporate CEOs (48 and 55%, respectively). Over time, such chronic pressure may gradually erode academics’ intrinsic interest, aspirations, and passion for scholarship, ultimately culminating in job burnout (Pace et al., 2021; Hammoudi Halat et al., 2023).

### 2.2 Job burnout in the academic profession and its determinants

Maslach (1976) first introduced the concept of burnout into the scientific literature, describing it as a gradual process of fatigue, cynicism, and reduced commitment among social care professionals. Building on subsequent empirical research, Maslach and his colleagues later refined the concept and developed the Maslach Burnout Inventory (MBI), which defines burnout in terms of high scores on emotional exhaustion and depersonalization, and low scores on personal accomplishment (Maslach and Jackson, 1981). This definition has since become the most influential and widely applied framework for understanding occupational burnout across diverse professions—including, increasingly, the academic sector (Roloff et al., 2022; Anastasiou, 2020; Siyum, 2023). As previously noted, the academic profession, characterized by its high levels of pressure and performance demands, is particularly susceptible to burnout and therefore warrants greater scholarly attention (Bozeman and Gaughan, 2011).

Regarding the determinants of job burnout in the academic profession, two classical frameworks, the Job Demands–Resources (JD-R) model (Bakker et al., 2014) and the person–organization/environment fit (PE-fit) model (Kristof, 1996), highlight that individual characteristics and organizational support represent the subjective (internal) and contextual (external) dimensions contributing to burnout, respectively. Building on these frameworks, perceived organizational support is conceptualized in this study as a core job resource that is directly associated with academic job burnout by alleviating strain and sustaining motivation under conditions of high and persistent job demands. At the same time, the PE-fit perspective highlights that the effectiveness of organizational resources depends on their alignment with individuals’ career needs and work contexts. Accordingly, career stage and professional sector are incorporated as structural conditions that may shape the strength of the association between perceived organizational support and job burnout. Rather than testing a single moderation mechanism, the present study adopts a conditional association approach, examining whether the support–burnout relationship varies systematically across career stages and professional sectors.

#### 2.2.1 Individual characteristics

The individual characteristics included in this study reflect dimensions most frequently examined in prior research on academic burnout, including gender, discipline, career stage, and professional sector.

##### 2.2.1.1 Gender

The prevailing view holds that female academics tend to experience higher levels of burnout than their male counterparts, largely due to implicit gender discrimination, work–family conflict, and unequal career opportunities (Tang and Horta, 2024; Rotenstein et al., 2021). However, Adekola (2010) found no significant gender differences in the levels of emotional exhaustion and depersonalization among university staff, while female employees reported significantly higher scores on reduced personal accomplishment compared with their male counterparts. This suggests that overall burnout levels may not systematically differ by gender, although women may experience a stronger sense of diminished accomplishment in the academic workplace.

##### 2.2.1.2 Discipline

Research on disciplinary variations in academic burnout remains limited. A cross-sectional study of 366 faculty members across applied human and social sciences, life and health sciences, and exact or technological sciences found no statistically significant differences in burnout or quality-of-life levels across disciplines (Alves et al., 2019).

##### 2.2.1.3 Professional sectors

Most existing research has focused on academics employed in higher education institutions, where faculty members often report heavy workloads and a heightened risk of burnout (Dewey et al., 2024). However, research on academic professionals working outside the university sector—such as in research institutes, government agencies, or industry—remains insufficient.

##### 2.2.1.4 Career stage

Whether job burnout varies across career stages has been a key focus of prior studies. Most scholars concur that early-career academics occupy a structurally disadvantaged position within the academic field. Confronted with multiple pressures—such as the “publish-or-perish” culture, promotion expectations, teaching and research demands, and family responsibilities—they are especially prone to burnout (Gibson, 2024; Kınıkoğlu and Can, 2021). Yet some studies have identified a “mid-career crisis” phenomenon, suggesting that burnout can also intensify during the middle stages of academic careers.

#### 2.2.2 Organizational support

Beyond individual characteristics, academics are embedded within organizational contexts, and their experience of burnout is often closely linked to the level of support they perceive from their institutions. As Khajuria and Khan (2022) argued, when employees feel valued, supported, and recognized by their organization, they tend to show greater engagement and motivation, whereas a lack of such support can lead to burnout. In the academic setting, Campbell and O'Meara (2014) highlighted that a positive institutional climate and access to adequate resources play an important role in fostering academic growth. Likewise, Clément et al. (2020) found that when institutions meet academics’ psychological needs and cultivate an atmosphere of trust, they can help sustain scholars’ energy, performance, and overall professional well-being.

## 3 Research questions and hypotheses development

Prior research has widely acknowledged the heightened risk of job burnout in the academic profession under conditions of heavy workload and performance pressure, and has examined how both individual and contextual factors (e.g., gender, discipline, career stage, and organizational support) relate to burnout. However, important gaps remain. First, much of the literature implicitly equates the academic profession with university faculty, paying limited attention to academic professionals working outside universities (e.g., research institutes, government agencies, and industry). Second, empirical evidence is often drawn from single countries or institutions, despite the increasingly global nature of academic burnout. Third, although organizational support has been repeatedly identified as a potentially important protective factor, less is known about the extent to which perceived organizational support is associated with burnout among academic professionals worldwide, and whether this association varies across career stages and professional sectors. To address these gaps, this study uses cross-national survey data and proposes the following research questions and hypotheses.

*RQ1*. What is the overall state and global pattern of job burnout in the academic profession?

*H1.* Job burnout is expected to be a widespread phenomenon in the academic profession

The global spread of performance-based management and intensified accountability in higher education has reshaped academic work across many contexts, making time scarcity and work overload increasingly common. Empirical surveys further indicate that academics are widely characterized by experiences of “time pressure” and “excessive workload,” and that their levels of job burnout tend to be higher than those observed in the general working population (Weinstein et al., 2023; Jerrim et al., 2021). These developments suggest that academic professionals, as a group, are likely to experience relatively high levels of job burnout. At the same time, national academic systems differ in employment security, evaluation regimes, and career structures (e.g., variations in tenure and promotion systems), which may generate cross-national differences in burnout levels.

*RQ2*. To what extent is perceived organizational support associated with academic job burnout?

*H2.* Perceived organizational support is negatively associated with academic job burnout

Drawing on the JD–R model (Bakker et al., 2014) and organizational support theory (Khajuria and Khan, 2022), perceived organizational support can be conceptualized as a key job resource in academic work contexts characterized by high and persistent demands. When academics believe that their organization values their contributions and cares about their well-being, supportive organizational conditions and practices can help buffer strain and sustain motivation, thereby being associated with lower levels of burnout.

*RQ3*. Does the association between perceived organizational support and academic job burnout vary across career stages and professional sectors?

*H3.* Career stage and professional sector independently moderate the association between perceived organizational support and academic job burnout

Drawing on the person–organization/environment fit perspective (Kristof, 1996), the effectiveness of organizational support depends on the extent to which organizational practices and resources align with individuals’ role expectations, career needs, and work contexts. Career stage reflects systematic differences in developmental needs, job security, and performance expectations, whereas professional sector captures distinct institutional logics and work structures that shape job demands and resource configurations. Consequently, the same level or type of perceived organizational support may not function equivalently across career stages or sectors. In line with the JD–R framework, which emphasizes that the salience of job resources is contingent on demand exposure and contextual conditions, we expect variation in the strength of the association between perceived organizational support and academic job burnout across career stages and professional sectors.

As illustrated in Figure 1, two complementary analytical models are specified. In the first model, career stage conditions the association between organizational support and job burnout, while controlling for gender, discipline, and professional sector. In the second model, professional sector conditions this association, while controlling for gender, discipline, and career stage. Together, these models allow for a clear and parsimonious examination of how organizational support operates as a key job resource, and how its association with burnout differs across structurally distinct academic contexts.

**Figure 1:**
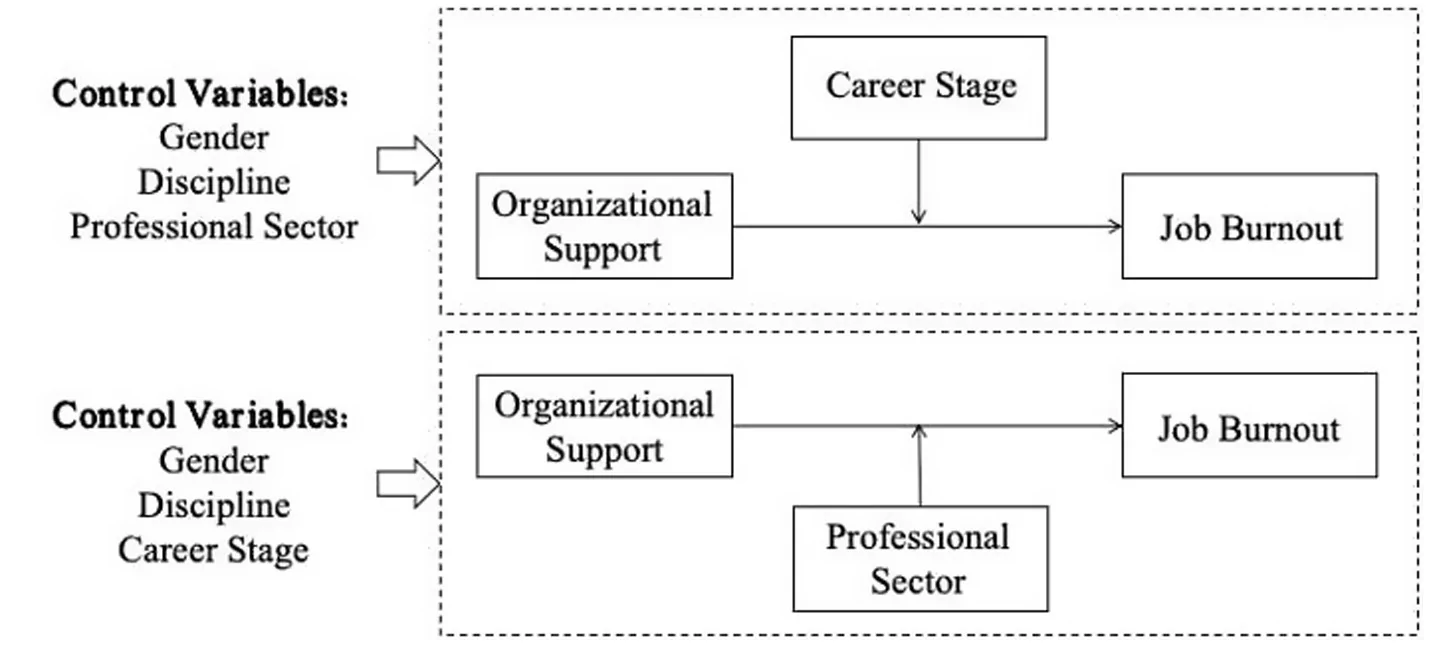
Hypothetical model of the moderating associations.

## 4 Method

### 4.1 Data source and participants

The data used in this study were drawn from the Nature Career Job and Salary Satisfaction Survey 2021, a large-scale international survey conducted to capture global trends in academic career satisfaction, income, and working conditions. The survey was distributed through Nature’s online platforms and mailing lists as an open and voluntary questionnaire targeting academic professionals worldwide. The questionnaire was administered in five languages, including English, Chinese, Spanish, French, and Portuguese, and yielded 3,210 valid responses from 95 countries. As the survey relied on open online recruitment rather than a predefined sampling frame, an exact response rate could not be calculated. After excluding respondents whose occupations were unrelated to academic or research work or whose career stages were unclear, and retaining only respondents with complete information on key variables, a total of 2,350 valid participants from 17 countries were included in the final analytical sample (see details in Table 1). These inclusion and exclusion criteria were applied to ensure the validity of the regression analyses rather than to achieve statistical representativeness. To further enhance analytical feasibility, only countries with a sample size greater than 30 were retained for regression analyses. This threshold was adopted to ensure sufficient within-country variation and stable parameter estimation and was not intended for descriptive cross-national comparisons. The number of respondents per country ranged from 47 to 892. Countries with sample sizes exceeding 100 included Australia (110), Canada (113), the United Kingdom (385), the United States (892), Spain (107), Germany (162), Brazil (105), and China (106). No weighting was applied in the analyses. Given the non-probability nature of the survey and the absence of reliable population benchmarks for the global academic workforce, applying post-stratification weights was not methodologically appropriate and could have introduced additional bias. In addition to quantitative data, this study also incorporated interview materials collected by Nature, which were used to supplement and contextualize the quantitative findings.

**Table 1:** Participants’ demographic.

Variable	Definition	Frequency (%)
Gender	0 = Female	1,100 (46.81%)
	1 = Male	1,193 (50.77%)
Discipline	1 = Science	662 (28.17%)
	2 = Engineering	459 (19.53%)
	3 = Medicine	1,062 (45.19%)
	4 = Humanities and social sciences	167 (7.11%)
Professional sector	0 = Non-academic sector	724 (30.81%)
	1 = academic Sector	1,626 (69.19%)
Career stage	1 = Early-stage	958 (40.77%)
	2 = Mid-stage	444 (18.89%)
	3 = Late-stage	948 (40.34%)

To assess the robustness of the findings, sensitivity analyses were conducted by excluding the largest country sample (the United States). The main regression results remained substantively unchanged in terms of direction and statistical significance, indicating that the findings were not driven by any single country.

### 4.2 Measurement

#### 4.2.1 Job burnout

Job burnout was measured using a multidimensional scale comprising three dimensions: emotional exhaustion (five items), depersonalization (three items), and reduced personal accomplishment (four items). Emotional exhaustion reflects a state of physical, emotional, and psychological fatigue caused by prolonged overwork (e.g., *I feel run down and drained of physical or emotional energy*). Depersonalization assesses the extent to which individuals display indifference, irritability, or detachment toward their work or colleagues (e.g., *I am easily irritated by my colleagues and team*). Reduced personal accomplishment captures individuals’ perceived lack of efficacy and self-worth in their professional context (e.g., *I feel unappreciated by my colleagues*). All items were rated on a five-point Likert scale, with higher scores indicating greater levels of burnout. Confirmatory factor analyses (CFA) were conducted to examine the factorial structure of job burnout. Two alternative models were tested and compared. The first was a correlated three-factor model, specifying emotional exhaustion, depersonalization, and reduced personal accomplishment as distinct but related first-order factors. This model demonstrated acceptable fit to the data (*χ^2^/df* = 18.08, CFI = 0.94, GFI = 0.94, IFI = 0.94, RMSEA = 0.09). The second was a second-order factor model, in which the three burnout dimensions loaded onto a higher-order latent burnout construct. This model showed slightly improved model fit (χ^2^/*df* = 15.14, CFI = 0.95, GFI = 0.95, IFI = 0.95, RMSEA = 0.08). Given its adequate fit and greater parsimony, the second-order model was retained to represent overall job burnout in subsequent analyses. The overall burnout scale demonstrated high internal consistency reliability (Cronbach’s *α* = 0.93).

#### 4.2.2 Perceived organizational support

Perceived organizational support was assessed using a seven-factor scale, each factor measured by three items on a five-point Likert scale. Consistent with the multidimensional conceptualization of organizational support, the seven dimensions were treated as distinct constructs in the subsequent analyses rather than being aggregated into a single overall index (*Cronbach’s α* = 0.93; *χ^2^/df* = 15.77, CFI = 0.90, GFI = 0.92, IFI = 0.90, RMSEA = 0.07). Collectively, these seven factors capture both instrumental and socio-emotional forms of organizational support, reflecting concrete organizational actions through which academics may experience being supported, valued, and recognized in their work. Specifically, instrumental support captures the provision of tangible resources and enabling conditions for academic work, including financial support (e.g., *availability of funding*), research support (e.g., *protected time for research*), career development support (e.g., *advancement opportunities*), administrative support (e.g., *management and leadership of the organization*), and workload conditions (e.g., *perceived total working hours*). Socio-emotional support reflects relational and affective resources that signal care, appreciation, and interpersonal connection, including interpersonal support (e.g., *guidance from supervisors*) and emotional support (e.g., *organizational concern for work meaningfulness and personal fulfillment*).

#### 4.2.3 Moderating factors

(1) Career stage was coded as an ordinal categorical variable, with participants identifying themselves as being in the early, mid, or late stage of their academic career. This self-reported measure reflects respondents’ perceived position within the academic career trajectory, capturing differences in employment security, evaluation pressure, role expectations, and accumulated resources. (2) Professional sector was coded as a binary variable, distinguishing between the academic sector (e.g., *university-based academic employment*) and the non-academic sector. The latter includes research-oriented positions outside universities, such as those in government agencies, industry, clinical settings, and non-profit organizations. This distinction is intended to capture differences in career structures and institutional logics, particularly whether academic work is embedded in traditional university-based promotion systems.

#### 4.2.4 Control variables

Individual characteristics, including gender and disciplinary field, were included as control variables in the analyses.

### 4.3 Analysis

The analysis proceeded in two stages. First, descriptive statistics (means and standard deviations) were used to summarize overall patterns of perceived organizational support and job burnout among academic professionals. To illustrate broad cross-national patterns, descriptive comparisons focused on selected countries with sample sizes greater than 100. This threshold was adopted to enhance the stability and interpretability of country-level mean estimates and to avoid over interpretation of descriptive patterns based on small national subsamples. Second, a series of linear regression models were estimated to examine the association between perceived organizational support and job burnout. All regression analyses were conducted using the full individual-level sample. To assess moderation, grouped regression models were employed, whereby separate models were estimated by career stage (early-, mid-, and late-career) and by professional sector (academic vs. non-academic). This approach allows the strength and significance of the associations between organizational support dimensions and job burnout to be compared across groups in a transparent manner. All models controlled for individual-level characteristics, including gender and discipline. Moderation was formally tested using multi-group structural equation modeling (SEM) by comparing constrained and unconstrained models. The results of these model comparisons are reported in Table 2. To facilitate interpretation of the moderation patterns, Table 3 presents subgroup-specific regression results for different career stages and professional sectors. Given the cross-national nature of the data, intraclass correlation coefficients (ICCs) were calculated to assess the degree of clustering by country. The ICCs were approximately 0.01, indicating that only about 1% of the variance in job burnout was attributable to between-country differences. Because the clustering effect was minimal, multilevel modeling was not pursued.

**Table 2:** Multi-group SEM results for moderation tests.

Moderating variables	Model	*χ^2^*	*df*	CFI	GFI	IFI	RMSEA
Career stages	Unconstrained	543.46	102	0.95	0.96	0.96	0.04
	Structural weights	599.04	120	0.95	0.96	0.96	0.04
	Model comparison	Δ*χ^2^* = 55.58; Δ*df* = 18; *p* = 0.000					
Professional sectors	Unconstrained	500.50	68	0.96	0.96	0.96	0.05
	Structural weights	545.49	89	0.96	0.96	0.96	0.05
	Model comparison	Δ*χ^2^* = 44.99; Δ*df* = 21; *p* = 0.002					

**Table 3:** Results of regression analyses for the full sample and subgroups.

Variable	Full sample (*n* = 2,350)	Early-career (*n* = 958)	Mid-career (*n* = 444)	Late-career (*n* = 948)	Non-Academic Sector (*n* = 724)	Academic Sector (*n* = 1,626)
Interpersonal support	−0.12 (0.02)^***^	−0.14(0.03)^***^	−0.18(0.05)^***^	−0.07(0.03) ^**^	−0.13(0.04) ^***^	−0.11(0.02) ^***^
Financial support	−0.05 (0.03)	−0.06(0.03)^*^	−0.02(0.04)	0.01(0.03)	−0.08(0.02) ^**^	−0.00(0.02)
Research support	−0.07 (0.02)^***^	−0.08(0.03)^*^	−0.01(0.04)	−0.08(0.03) ^**^	0.00(0.03)	−0.10(0.02) ^***^
Career development support	−0.04 (0.02)^*^	−0.06(0.03)^*^	−0.07(0.05)	−0.01(0.03)	−0.00(0.04)	−0.09(0.02) ^***^
Organizational Management support	−0.14(0.02)^***^	−0.14(0.03)^***^	−0.18(0.04)^***^	−0.14(0.03) ^***^	−0.13(0.04) ^***^	−0.13(0.03) ^***^
Emotional support	−0.14(0.02)^***^	−0.12(0.02)^***^	−0.17(0.04)^***^	−0.15(0.03) ^***^	−0.16(0.03) ^***^	−0.15(0.02) ^***^
Workload conditions	−0.15(0.02)^***^	−0.09(0.02)^***^	−0.17(0.04)^***^	−0.20(0.03) ^***^	−0.16(0.04) ^***^	−0.14(0.02) ^***^
Gender	−0.19(0.03)^***^	−0.22(0.04)^***^	−0.20(0.06)^***^	−0.15(0.04) ^***^	−0.23(0.05) ^***^	−0.19(0.03) ^***^
Discipline	Controlled					
Career stage (mid vs. early)	−0.29(0.04)^***^	—			Controlled	
Career stage (late vs. early)	−0.07(0.03)^**^	—			Controlled	
Professional sector	0.10(0.02)^***^	Controlled			—	
Constant	5.23(0.08)^***^	5.31(0.12)^***^	4.93(0.20)^***^	5.07(0.12)^***^	5.08(0.13) ^***^	5.31(0.09) ^***^
Adjusted *R^2^*	0.41	0.41	0.36	0.39	0.38	0.39
*F*	113.58^***^	54.10^***^	21.71^***^	49.31^***^	40.49^***^	90.89^***^

Entries are unstandardized coefficients with standard errors in parentheses; *t* statistics can be obtained as B/SE. Degrees of freedom are determined by subgroup sample size and model specification. ^***^p < 0.001, ^**^p < 0.01, ^*^p < 0.05.

## 5 Results

### 5.1 Overview of organizational support and job burnout among academics

Academics generally reported experiencing job burnout, characterized by reduced personal accomplishment (*M* = 3.02, *SD* = 0.91) and emotional exhaustion (*M* = 2.83, *SD* = 0.90), and were often prone to negative emotional responses toward their work and colleagues (*M* = 2.47, *SD* = 0.87). Most organizations were found to provide relatively comprehensive support services for academic professionals (*M* = 3.47, *SD* = 0.70). Respondents expressed the highest satisfaction with emotional (*M* = 3.93, *SD* = 1.02) and research support (*M* = 3.66, *SD* = 1.02). In contrast, lower satisfaction was reported in areas such as financial support (*M* = 3.22, *SD* = 0.91), career development support (*M* = 3.20, *SD* = 0.93), and organizational management (*M* = 3.14, *SD* = 0.91). Notably, a comparison between the *Nature*’s surveys conducted in 2018 and 2021 showed a sharp increase in the proportion of respondents who were “dissatisfied” or “very dissatisfied” with their academic work—from 18 to 34%. Moreover, more than half of the 2021 respondents (54%) reported lacking sufficient organizational support over the past year. Some respondents highlighted their frustrations, stating that “The culture of science today is fundamentally inhuman and demoralizing,” “We are already an overworked and underpaid sector, especially considering our high level of education,” and that “The government never took action” (Woolston, 2021a, 2021b). Overall, these descriptive results are consistent with H1, which anticipated the widespread nature of job burnout in the academic profession.

The study further compared differences in job burnout and perceived organizational support across career stages and professional sectors. The results revealed significant variations in overall burnout levels among groups (*p* < 0.01). Specifically, burnout followed the pattern early-career > late-career > mid-career, and was higher among academics working in academia than those outside academia. In terms of satisfaction with organizational support, the pattern was reversed: mid-career > early-career > late-career, and non-academic > academic sectors. Qualitative evidence from the *Nature* interviews supported these results. For example, an assistant professor remarked, “I am very concerned about the huge negative impact on early-career researchers, who were unable to get papers out or to generate data for grant applications at this crucial stage in our careers.” Another respondent commented that “Everyone’s skills are really valued… [In industry], I wasn’t spread between three or four tasks in a given day,” in stark contrast to his previous experience in the university setting (Woolston, 2021a, 2021b).

### 5.2 Cross-national patterns in organizational support and job burnout

Descriptive cross-national patterns were observed in academics’ perceptions of organizational support and job burnout across selected countries, providing an illustrative overview of how these experiences vary internationally, which is consistent with H1. First, academic professionals in China and Brazil reported comparatively lower levels of satisfaction with organizational support; however, their reported burnout levels differed markedly. China exhibited relatively low average levels of job burnout, whereas Brazil showed comparatively high levels. Second, academic professionals in Canada and Germany tended to report higher levels of organizational support alongside lower average burnout levels. Third, although respondents in the United States and Australia reported above-average levels of organizational support, they also reported relatively elevated levels of burnout. These descriptive patterns are summarized in Figure 2.

**Figure 2:**
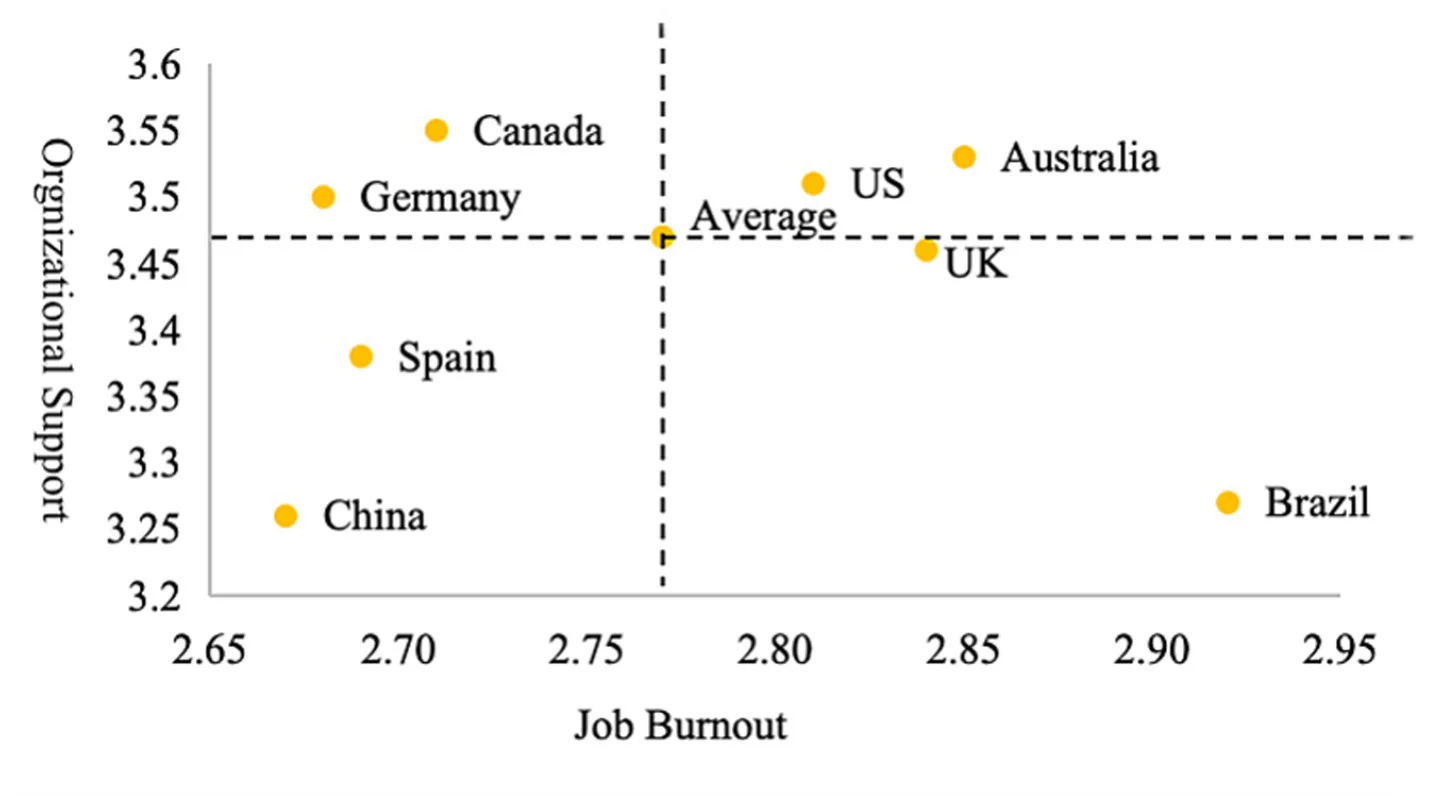
Descriptive cross-national variation in organizational support and job burnout.

In terms of organizational support, academics in China reported comparatively lower average scores across most support dimensions, particularly interpersonal support, emotional support, and workload management. Financial, research, and organizational management support were also rated relatively lower. By contrast, respondents in countries such as Australia, Germany, the United States, and Canada reported higher average levels across multiple dimensions of organizational support. For example, Australian respondents reported relatively higher ratings for interpersonal, research, and emotional support; German academics reported higher financial and emotional support; U. S. respondents expressed greater satisfaction with career development opportunities; and Canadian respondents highlighted comparatively better organizational management and workload balance. These patterns are illustrated in Figure 3.

**Figure 3:**
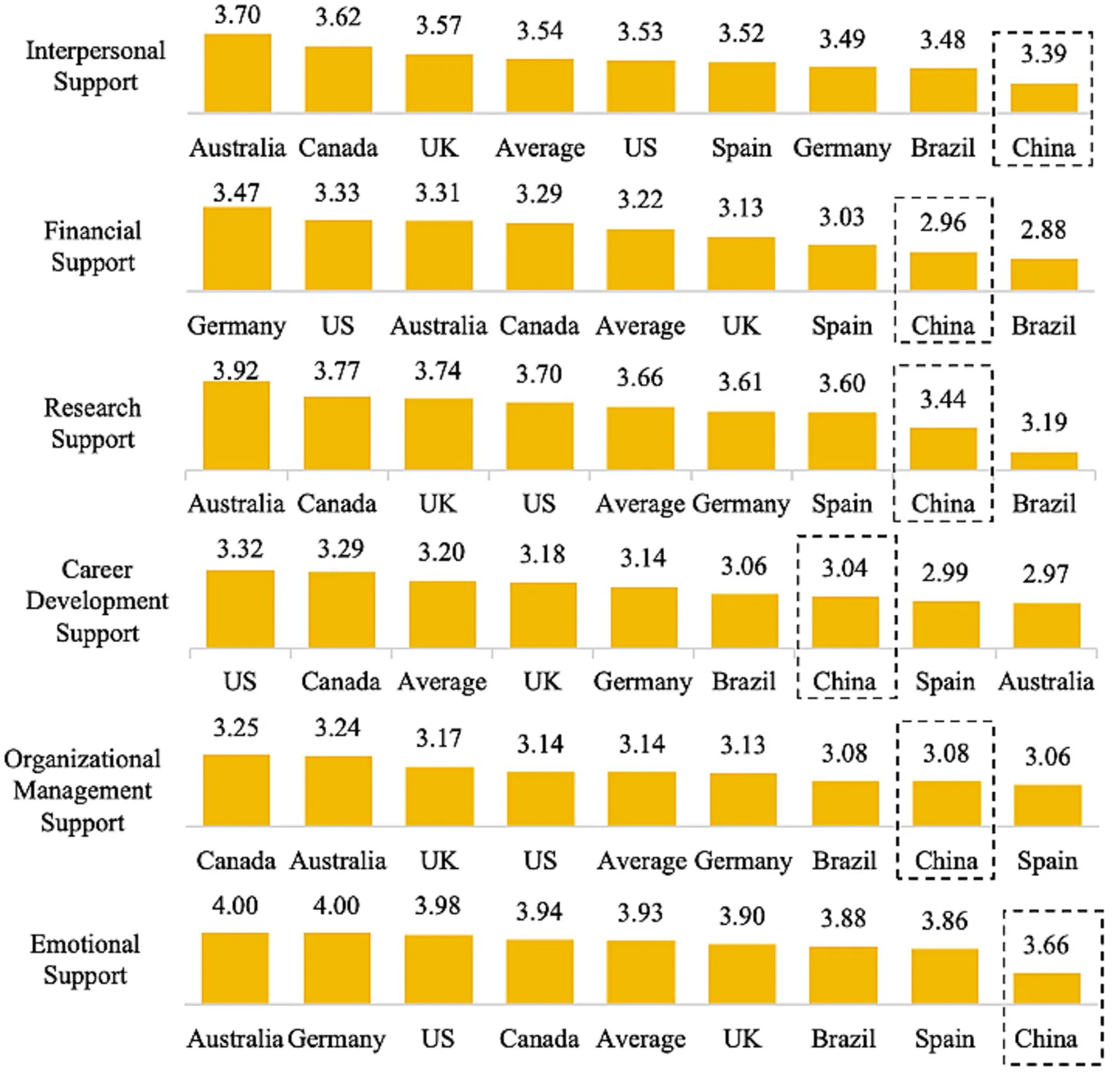
Descriptive cross-national variation in organizational support.

### 5.3 Moderating association between career stage and professional sector

The study conducted multiple regression analyses using the full sample, as well as subsamples divided by career stage and professional sector. The regression models were statistically significant (*p* < 0.001), explaining between 36 and 41% of the variance in job burnout, with no evidence of multicollinearity among predictors (VIF < 2). To formally examine moderation, multi-group structural equation modeling (SEM) was conducted using AMOS 22.0. Moderation was tested by comparing constrained and unconstrained models across career stages and professional sectors, with differences in model fit (Δ*χ^2^*, Δ*df*) used to assess the significance of moderation associations. The results of these model comparisons are reported in Table 2.

Overall, interpersonal support, research support, career development support, organizational management support, emotional support, and workload management were significantly associated with lower levels of job burnout among academics. These findings are consistent with H2, which posited a negative association between perceived organizational support and academic job burnout. In addition, the multi-group SEM results indicate that the strength of these associations varies across career stages and professional sectors, providing evidence consistent with H3 regarding moderation in the association. Specifically, the chi-square difference tests indicated that constraining the structural paths significantly worsened model fit across both career stages (Δ*χ^2^* = 55.58, Δ*df* = 18, *p* < 0.001) and professional sectors (Δ*χ^2^* = 44.99, Δ*df* = 21, *p* < 0.01), indicating significant moderation in the association between perceived organizational support and job burnout. To facilitate interpretation of these moderation patterns, Table 3 presents subgroup-specific regression results for different career stages and professional sectors. Results from robustness checks using cluster-robust standard errors at the country level are reported in Table 4. The results are substantively identical to those reported in the main analyses.

**Table 4:** Robustness checks using cluster-robust standard errors (cluster = country).

Variable	Full sample (*n* = 2,350)	Early-career (*n* = 958)	Mid-career (*n* = 444)	Late-career (*n* = 948)	Non-Academic Sector (*n* = 724)	Academic Sector (*n* = 1,626)
Interpersonal support	−0.12(0.02)^***^	−0.14(0.02)^***^	−0.18(0.04)^***^	−0.07(0.03) ^**^	−0.13(0.04) ^***^	−0.13(0.03) ^***^
Financial support	−0.02(0.01)	−0.06(0.03)^*^	−0.02(0.03)	0.01(0.03)	−0.07(0.02) ^**^	−0.02(0.02)
Research support	−0.07(0.02)^***^	−0.08(0.02)^**^	−0.01(0.03)	−0.08(0.02) ^***^	0.01(0.03)	−0.10(0.02) ^***^
Career development support	−0.04(0.02)^*^	−0.06(0.03)^*^	−0.07(0.05)	−0.01(0.03)	−0.00(0.04)	−0.09(0.02) ^***^
Organizational management support	−0.14(0.02)^***^	−0.14(0.02)^***^	−0.15(0.03)^***^	−0.14(0.03) ^***^	−0.14(0.03) ^***^	−0.14(0.03) ^***^
Emotional support	−0.14(0.02)^***^	−0.12(0.02)^***^	−0.17(0.04)^***^	−0.15(0.03) ^***^	−0.15(0.02) ^***^	−0.15(0.02) ^***^
Workload conditions	−0.15(0.02)^***^	−0.09(0.02)^***^	−0.17(0.05)^**^	−0.20(0.02) ^***^	−0.16(0.02) ^***^	−0.16(0.02) ^***^
Gender	−0.19(0.02)^***^	−0.22(0.04)^***^	−0.20(0.04)^***^	−0.15(0.05) ^*^	−0.22(0.03) ^***^	−0.22(0.03) ^***^
Discipline	Controlled					
Career stage (mid vs. early)	−0.29(0.03)^***^	—			Controlled	
Career stage (late vs. early)	−0.06(0.02)^**^	—			Controlled	
Professional sector	0.08(0.02)^***^	Controlled			—	
Constant	5.23(0.06)^***^	5.31(0.10)^***^	4.93(0.18)^***^	5.07(0.11)^***^	5.15(0.10) ^***^	5.15(0.10) ^***^
Adjusted *R^2^*	0.41	0.41	0.38	0.39	0.40	0.40
*F*	2908.28^***^	238.78^***^	221.55^***^	2651.69^***^	503.29^***^	503.29^***^

^***^p < 0.001, ^**^p < 0.01, ^*^p < 0.05.

At the early-career stage, academics reported the strongest associations between perceived organizational support and lower burnout across all dimensions. Among these, interpersonal support, organizational management support, and emotional support showed the strongest associations with burnout levels. These patterns suggest that multiple forms of organizational support are particularly salient for early-career academics. In the mid-career stage, burnout was most strongly associated with interpersonal, organizational, and emotional support, as well as workload management, whereas the associations between financial, research, and career development support and job burnout were relatively weaker. For late-career academics, workload management showed the strongest association with burnout, followed by organizational management and emotional support. In contrast, financial and career development support were not significantly associated with burnout levels in this group.

Regarding professional sectors, burnout among non-academic professionals was more strongly associated with emotional support, workload management, interpersonal support, organizational management, and financial support, while research and career development support showed weaker or non-significant associations. As one U.S. researcher who worked for the government noted, “Improve the culture, the job security, stabilize funding, and then people with talent will flock to science” (Woolston, 2021a). By contrast, academics working within universities showed stronger associations between burnout and research and career development support, whereas financial support was not significantly associated with burnout levels in this group.

The analysis also revealed that female academics reported significantly higher levels of burnout than their male counterparts, whereas no significant differences were observed across disciplines. As one U.S. female academic reflected, during the early stages of her career, she had to invest substantially more “time, effort, and personal resources” than her male colleagues in order to obtain comparable levels of funding and professional recognition (Woolston, 2021a). Many female scholars reported feeling exhausted and demotivated due to a lack of adequate recognition for their achievements.

## 6 Discussion and implications

### 6.1 General discussion

#### 6.1.1 The pervasiveness and group heterogeneity of academic burnout

Job burnout was found to be a prevalent phenomenon among academics, characterized by varying degrees of emotional exhaustion, depersonalization, and reduced personal accomplishment. The findings further revealed clear group heterogeneity: female academics and those in the early stages of their careers reported significantly higher levels of burnout, which is consistent with prior research (Tang and Horta, 2024; Gibson, 2024). First, female scholars often experience a dual burden of professional and family responsibilities, as well as implicit gender bias within academia. Such factors tend to slow their career progression and make them more vulnerable to exhaustion under comparable workloads (Ellis, 2021). Second, early-career academics face a convergence of multiple, compounding pressures—including financial insecurity, family obligations, and academic pressure—exacerbated by the uncertainty of “up-or-out” employment systems (Park et al., 2024). Moreover, the so-called “mid-career crisis” observed in other studies was not supported by the present findings (Woolston, 2021a). On the contrary, mid-career academics displayed the highest satisfaction with organizational support and the lowest levels of burnout. Third, academics in university settings exhibited higher burnout than those working in non-academic sectors. While non-academic professionals are typically focused on research and development tasks, university faculty members must balance teaching, research, and service responsibilities, leading to heavier workloads and greater role strain (Hammoudi Halat et al., 2023). Many university respondents also noted that lower pay compared to their industry peers, combined with the job insecurity associated with performance evaluation and tenure-track systems, further exacerbated their burnout.

#### 6.1.2 Divergent national configurations of organizational support and burnout

Cross-national comparisons reveal four distinct configurations of organizational support and job burnout among academic professionals. China and Brazil both exhibited relatively low levels of organizational support; however, the two countries stood at opposite ends of the burnout spectrum, with China reporting the lowest and Brazil the highest levels of burnout. In contrast, the United States showed relatively high organizational support but still experienced elevated burnout levels. Germany and Canada, meanwhile, represented a more favorable pattern—high organizational support accompanied by low burnout.

A closer examination suggests that these patterns are closely linked to the stability of national academic employment systems. In the United States, an oversaturated academic labor market combined with intensifying performance-based accountability has reshaped academic employment structures. Many institutions increasingly rely on non-tenure-track and part-time appointments, leaving scholars exposed to ongoing job insecurity and limited long-term prospects (Allen and Sweeney, 2017). By contrast, although some Chinese universities have recently adopted “up-or-out” systems, the academic labor market overall remains relatively closed and less competitive (Wang and Wang, 2024). Once appointed, many academics effectively enter a quasi-tenure track, enjoying comparatively strong employment stability. These institutional contrasts indicate that greater job stability may be associated with lower levels of burnout, whereas precarious employment amplifies stress and emotional exhaustion. Nevertheless, in countries such as China and Spain, where burnout levels are comparatively low, the lack of adequate organizational support may hinder sustained professional growth and intrinsic motivation. As more universities in these countries implement “up-or-out” mechanisms, rising psychological isolation and uncertainty could gradually erode academics’ sense of purpose and meaning, potentially triggering a new wave of burnout within the academic profession.

#### 6.1.3 Heterogeneity in the association between perceived organizational support and job burnout

Before interpreting the associations between perceived organizational support and academic job burnout and its heterogeneity across groups, it is necessary to clarify what perceived organizational support represents in this study and how it may operate. Grounded in organizational support theory and a social exchange perspective, perceived organizational support refers not merely to the objective resources an organization provides, but to an overarching belief formed by employees that the organization values their contributions and cares about their well-being—and that, on this basis, it is willing to offer various forms of support and protection through institutional arrangements and everyday practices (Khajuria and Khan, 2022). Such perceptions of being valued and supported encourage employees to invest more fully in their work and display higher levels of engagement (Aktar and Pangil, 2018). For academics, feeling “supported, recognized, and valued” typically draws on two complementary sets of cues: instrumental cues (e.g., adequate resources, manageable workload conditions, and administrative arrangements) that enhance the sense of being able to meet job demands and reduce strain, and socio-emotional cues (e.g., affirmation, respect, care, and support for work meaningfulness) that signal appreciation and strengthen belonging and motivation. Accordingly, the seven-domain perceived organizational support measure used here can be understood as observable organizational practices and contextual features that function as key job resources, through both strain reduction and motivation support, providing a conceptual foundation for the following discussion of variations across career stages and professional sectors.

The finding that perceived organizational support is associated with lower job burnout among academics is consistent with prior research (Khajuria and Khan, 2022). This pattern also aligns with the JD–R model, which distinguishes between job demands that deplete energy and job resources that sustain motivation and engagement (Bakker et al., 2014). For academics, job demands are multifaceted and persistent, encompassing responsibilities related to research, teaching, administration, and service (Bentley and Kyvik, 2012). In addition, the academic profession is characterized by long-term uncertainty, intense competition, and sustained pressure, making perceived organizational support a crucial job resource that may mitigate strain and foster engagement.

This study further indicates that the strength of this association varies across career stages and professional sectors. In the early-career stage, academics may be particularly vulnerable due to insecure employment and limited access to institutional resources. Comprehensive and multidimensional support can therefore be important for sustaining motivation, retention, and professional growth (Lee, 2024); conversely, limited support may be linked to chronic stress that undermines persistence and career progression (Benz et al., 2021). In contrast, mid- and late-career academics often exhibit greater stability and professional confidence as research achievements accumulate (Baker and Manning, 2020). Their reliance on financial, research, and career-development support may gradually diminish over time. Yet, as health concerns, fatigue, and career stagnation become more salient later in the career (Howson, 2025), interpersonal support, symbolic recognition, and work–life balance may become increasingly important for sustaining motivation and academic vitality.

Furthermore, the observed differences across professional sectors shed light on the structural conditions that shape support–burnout relationships. Academics working in university settings face constant pressure related to research productivity, reputation, and promotion expectations. Consequently, they may place greater weight on research and career-development support to sustain progress in competitive environments where scholarly output and advancement opportunities are key indicators of success. By contrast, those employed in non-academic sectors (e.g., government agencies and industry) often work in project-oriented systems and may prioritize financial and socio-emotional support to maintain short-term performance and job security. Adequate funding can enhance efficiency and stability, whereas emotional support may help offset uncertainty and stress in performance-driven and flexible employment arrangements, thereby strengthening belonging within the organization.

### 6.2 Practical implications

#### 6.2.1 Recognizing the early warning function of academic burnout

The present findings underscore the importance of treating academic burnout as an early warning signal of systemic strain within research organizations. Academic work has long been associated with intrinsic curiosity, creativity, and the pursuit of truth. To sustain these values, institutions need to foster an environment that balances academic rigor with psychological well-being and autonomy. Rather than relying on rigid, metric-driven performance evaluation systems, institutions may benefit from adopting more flexible and individualized assessment practices that consider disciplinary differences, research orientations, and the inherent risks of innovation. Such approaches can promote fairness and protect intrinsic motivation among academics. Likewise, minimizing excessive administrative control and bureaucratic demands, such as teaching-related paperwork, grant management, or travel approvals, can reduce unnecessary stress and allow faculty greater autonomy in shaping their work. Particular attention should be given to populations most vulnerable to burnout, including female scholars, early-career academics, and those working within university settings. Targeted interventions that provide mentorship, equitable evaluation, and accessible organizational resources may help mitigate burnout and support the long-term professional growth of these groups.

#### 6.2.2 Building a structured system of organizational support

Alleviating job burnout within the academic profession requires a comprehensive, structured, and responsive organizational support mechanism. First, financial and administrative support are fundamental. Organizations need to optimize the allocation of research resources rather than linking compensation and opportunities solely to titles or formal distinctions. Particular attention should be given to early-career scholars, who would benefit from initiatives such as independent innovation funds or start-up research grants designed to facilitate their integration into academic careers. Second, interpersonal and emotional support should be strengthened. Institutions can provide access to professional counseling and mentoring in psychological, academic, and personal domains, while also fostering peer-support communities. Third, research and career-development support play a critical role in sustaining long-term engagement. Drawing inspiration from international models of “academic communities,” institutions can cultivate diverse and collaborative environments by organizing research salons, thematic workshops, and mentoring programs. These initiatives can provide guidance on grant applications, promotion processes, and career planning, thereby enhancing both scholarly productivity and career satisfaction. Finally, organizations can leverage digital platforms to maintain ongoing communication with academic staff, collecting feedback and identifying stressors in real time.

#### 6.2.3 Providing targeted support across career stages and professional sectors

Organizational support should be tailored to the diverse needs of academics across career stages and professional sectors. For early-career scholars, targeted mechanisms such as mentorship programs, start-up grants, and research sabbaticals can facilitate adaptation to academic norms and the development of research independence. Structured guidance and protected research time may also buffer stress and prevent early burnout. For mid- and late-career academics, strategies should focus on maintaining vitality and fostering creativity. Streamlined administrative processes, flexible work arrangements, and opportunities for leadership or recognition can sustain motivation and promote intellectual renewal. Support systems should also differ across professional sectors. University-based scholars rely heavily on research and career-development resources; thus, stable funding, adequate laboratory facilities, and transparent promotion pathways are essential. In contrast, academic professionals working in industry or government often prioritize financial stability and emotional well-being. Dedicated project funds, peer-support networks, and access to mental health services can mitigate stress from performance pressure or job insecurity while strengthening their sense of belonging and professional security.

## 7 Conclusion

Weber (2012) famously described the academic career as “a reckless adventure.” More than a century later, his words remain strikingly relevant. Under the growing pressures of new managerialism, the academic profession faces an increasing risk of job burnout—a collective manifestation of systemic strain that extends far beyond individual distress. Can organizational support serve as a protective buffer, alleviating the exhaustion that permeates academic life? This study sought to address this question by examining the association between organizational support and job burnout among academics across different career stages and professional sectors. Drawing on global survey data, the results provide strong empirical evidence of a significant association between organizational support and lower levels of burnout, though the associations vary by context. These findings offer valuable insights for improving institutional practices and promoting sustainable academic careers.

At the same time, this study has several limitations that suggest directions for future research. First, the analyses were based on cross-sectional survey data, which limits causal inference. Future research may employ longitudinal designs or more rigorous causal inference approaches to further examine these relationships. Second, all key variables were measured using self-reported data, which may be subject to common method variance or response bias. In addition, participation in the survey was voluntary, raising the possibility that academics experiencing higher levels of stress or psychological distress were more likely to respond, which may affect population representativeness and the estimation of burnout prevalence. Future studies could incorporate multi-source data or objective indicators to reduce potential bias. Third, while several mechanisms were proposed to explain differences across career stages and professional sectors, such as employment security, evaluation pressures, and institutional logics, these mechanisms were not directly tested in the empirical models. Future research should examine these explanatory pathways more explicitly. Fourth, the reported cross-national differences were descriptive in nature, as no inferential statistical tests were conducted to compare country-level effects. Future research may adopt multilevel or comparative designs to investigate cross-national variation more rigorously.

## Data Availability

Publicly available datasets were analyzed in this study. This data can be found at: https://figshare.com/articles/dataset/Nature_Careers_job_and_salary_satisfaction_survey_2021/16860031?file=31323085.
